# Impact of preoperative physical activity and depressive symptoms on post-cardiac surgical outcomes

**DOI:** 10.1371/journal.pone.0213324

**Published:** 2019-02-28

**Authors:** D. Scott Kehler, Andrew N. Stammers, David Horne, Brett Hiebert, George Kaoukis, Todd A. Duhamel, Rakesh C. Arora

**Affiliations:** 1 Health, Leisure & Human Performance Research Institute, Faculty of Kinesiology and Recreation Management, University of Manitoba, Winnipeg, Manitoba, Canada; 2 Institute of Cardiovascular Sciences, St. Boniface Hospital Research Centre, Winnipeg, Manitoba, Canada; 3 Section of Cardiac Surgery, IWK Health Centre, Dalhousie University, Halifax, Nova Scotia, Canada; 4 Department of Surgery, Max Rady Faculty of Health Sciences, University of Manitoba, Winnipeg, Manitoba, Canada; 5 St. Boniface General Hospital, Cardiac Psychology Service, Winnipeg, Manitoba, Canada; Shenandoah University College of Arts and Sciences, UNITED STATES

## Abstract

**Objective:**

To determine the independent and combined impact of preoperative physical activity and depressive symptoms with hospital length of stay (HLOS), and postoperative re-hospitalization and mortality in cardiac surgery patients.

**Methods:**

A cohort study including 405 elective and in-house urgent cardiac surgery patients were analyzed preoperatively. Physical activity was assessed with the International Physical Activity Questionnaire to categorize patients as active and inactive. The Patient Health Questionnaire-9 was used to evaluate preoperative depressive symptoms and categorize patients as depressed and not depressed. Patients were separated into four groups: 1) Not depressed/active (n = 209), 2) Depressed/active (n = 48), 3) Not depressed/inactive (n = 101), and 4) Depressed/inactive (n = 47). Administrative data captured re-hospitalization and mortality data, and were combined into a composite endpoint. Models adjusted for demographics, comorbidities, and cardiac surgery type. Multiple imputation was used to impute missing values.

**Results:**

Preoperative physical activity behavior and depression were not associated with HLOS examined in isolation or when analyzed by the physical activity/depressive symptom groups. Physical inactivity (HR: 1.60, 95% CI 1.05 to 2.42; p = 0.03), but not depressive symptoms, was independently associated with the composite outcome. Freedom from the composite outcome were 76.1%, 87.5%, 68.0%, and 61.7% in the Not depressed/active, Depressed/active, Not depressed/inactive, and Depressed/inactive groups, respectively (P = 0.02). The Active/Depressed group had a lower risk of the composite outcome (HR: 0.35 95% CI 0.14 to 0.89; p = 0.03) compared to the other physical activity/depression groups.

**Conclusion:**

Preoperative physical activity appears to be more important than depressive symptoms on short-term postoperative re-hospitalization and mortality.

## Introduction

Cardiac surgery seeks to improve cardiac symptoms, quality of life and long-term survival in patients with significant coronary artery and valvular disease. However, non-traditional risk factors, such as elevated depressive symptoms, are highly prevalent in the cardiac surgery population, which could negatively impact their health outcomes peri-operatively despite a technically successful procedure [1]. Contemporary studies estimate that 20–38% of cardiac surgery patients are depressed in the peri-operative setting which is considerably higher than the general population and in patients with coronary artery disease not requiring surgical intervention [2–5]. Preoperative depression and depressive symptoms appear to be independent risk factors for poor outcomes, such as mortality, hospital readmissions, recurrent cardiac events, and poor health-related quality of life [3, 6, 7], which may persist in the postoperative period even though the surgical procedure was uneventful [8]. In response to these important investigations and based on the recommendations from the American Heart Association [9], there is an increased interest in implementing routine depression screening in clinical practice in an attempt to identify depressed cardiac surgery patients preoperatively, although data to support this recommendation are limited [10].

Plausible behavioral factors have been proposed to explain the association between depression and poor cardiovascular outcomes, including physical activity behavior [11, 12]. Evidence suggests that the combination of physical inactivity and clinically elevated depressive symptoms are more detrimental than either in isolation for cardiac mortality, but physical activity behavior may be more strongly associated with poor health outcomes than depression [12, 13]. Given the modest reductions in depressive symptoms from first line treatment strategies such as antidepressants in patients with coronary artery disease, some clinicians and patients have sought alternative therapeutic approaches, such as exercise therapy [10, 14]. Indeed, previous studies have shown that preoperative exercise rehabilitation strategies can shorten hospital length of stay (HLOS) and improve postoperative functional capacity and quality of life among younger elective patients undergoing coronary artery bypass [15, 16]. Our previous study also demonstrated that a more physically active lifestyle before cardiac surgery was independently associated with a reduction in the prevalence of depressive symptoms [5]. However, what is missing from these investigations are no analyses of the impact of pre-surgical physical activity behaviors and depressive symptoms, either in combination or in isolation, on operative and postoperative outcomes in patients undergoing elective or in-house urgent cardiac surgery. Here, we examine the associations between preoperative physical activity levels and depressive symptoms on HLOS and one-year re-hospitalization and mortality rates.

## Materials and methods

This study was a post-hoc analysis from a prospective observational study of 436 consecutively consenting patients undergoing elective or in-house urgent cardiac surgery in Canada [4, 5]. The study was supported by an unrestricted operating grant from Pfizer Canada. This organization was not involved in the study design, collection of data, or analysis and interpretation of the study findings, nor had the right to approve or disprove the manuscript. The study protocol was approved by the University of Manitoba Human Research Ethics Board, the St. Boniface Hospital Research Review Committee, and the Manitoba Health Information Privacy Committee.

### Patients

All patients provided informed consent and were recruited into the study from May 2010 through August 2011. Patients ≥18 years of age who could understand English and underwent coronary artery bypass graft (CABG), valve repair/replacement, or combined CABG and valve procedures, were recruited. Patients with cognitive impairment or those requiring an operative HLOS greater than three months were excluded from participating in the study. Recruitment, consent, and questionnaires were collected from elective patients two weeks prior to their procedure at their pre-assessment appointment with an Anesthetist and Nurse Practitioner, while semi-urgent patients underwent recruitment, consent, and data collection 1–3 days prior to their operation while they were stabilized in hospital for a cardiac event. Data collection occurred at these time points.

### Physical activity

Physical activity behavior was captured preoperatively using the short-form International Physical Activity Questionnaire (IPAQ). The IPAQ has been validated across 12-countries and performs similar to other self-report measures in patients with cardiovascular disease [17, 18]. Patients were classified as “active” or “inactive” based on accumulating 600 MET-min/week of physical activity, which corresponds to walking approximately 30 minutes, 5 days per week.

### Depression

Preoperative depressive symptoms were evaluated with the self-administered 9-item Patient Health Questionnaire-9 (PHQ-9) [19]. The PHQ-9 is a reliable and valid measure of depression severity in adults over the age of 18 and in patients hospitalized for coronary artery disease [19]. The PHQ-9 examines the frequency of depressive symptoms based on the Diagnostic Statistical Manual of Mental Disorders over the preceding two-week period from not at all, several days, more than half the days, or nearly every day (scores range from 0–27) [20]. Patients with elevated depressive symptoms were defined in three ways: 1) scoring >4 on the PHQ-9 with more than three positive responses, 2) a PHQ-9 score of 5–9 plus a risk factor for depression, including family history, living alone, previously diagnosed depression, currently controlled using antidepressants, and a recent stressful event, and 3) a PHQ-9 score of ≥10. This method of depression classification has been used previously in the cardiac surgery population [4].

### Outcomes

To investigate HLOS after cardiac surgery and one-year postoperative re-hospitalization and mortality, de-identified patient data was linked to administrative and clinical databases populated within the Manitoba Centre for Health Policy. Data housed within this centre have been previously validated [21]. Clinical data and hospital outcomes were linked with depression and physical activity data from the research database. Additionally, administrative data were linked with research data to collect Vital Statistics Mortality, and re-hospitalizations to provincial hospitals.

### Statistical analysis

Continuous variables were compared using a Kruskal Wallis Test and categorical variables were compared using a Chi-Square Test. Predictors of HLOS for a patient's initial surgical hospitalization for cardiac surgery were assessed via multivariable linear regression with a log transformation. Due to a small number of mortality rates between groups, a composite endpoint was developed to assess outcomes one year postoperatively, which included mortality or re-admission to a local hospital within one year of initial discharge following cardiac surgery. A multivariable proportional hazards regression model was developed to identify if clinical characteristics, physical activity, or depressive symptoms, were associated with this outcome. Variables for all regression analyses were inputted into the model based on their clinical relevance. A Kaplan Meier Curve was developed to visualize the differences in the one year composite outcome for people dichotomized into depressed/not depressed and physically active active/inactive. The analyses described above were re-run to evaluate the combined impact of preoperative depressive symptoms and physical activity. This process was completed by stratifying patients into four groups: 1) Not depressed/Active, 2) Depressed/Active, 3) Not depressed/Inactive, and 4) Depressed/Inactive based on their PHQ-9 and IPAQ score definitions. Missing values were imputed using multiple imputation.

## Results

### Baseline characteristics

**Fig 1:**
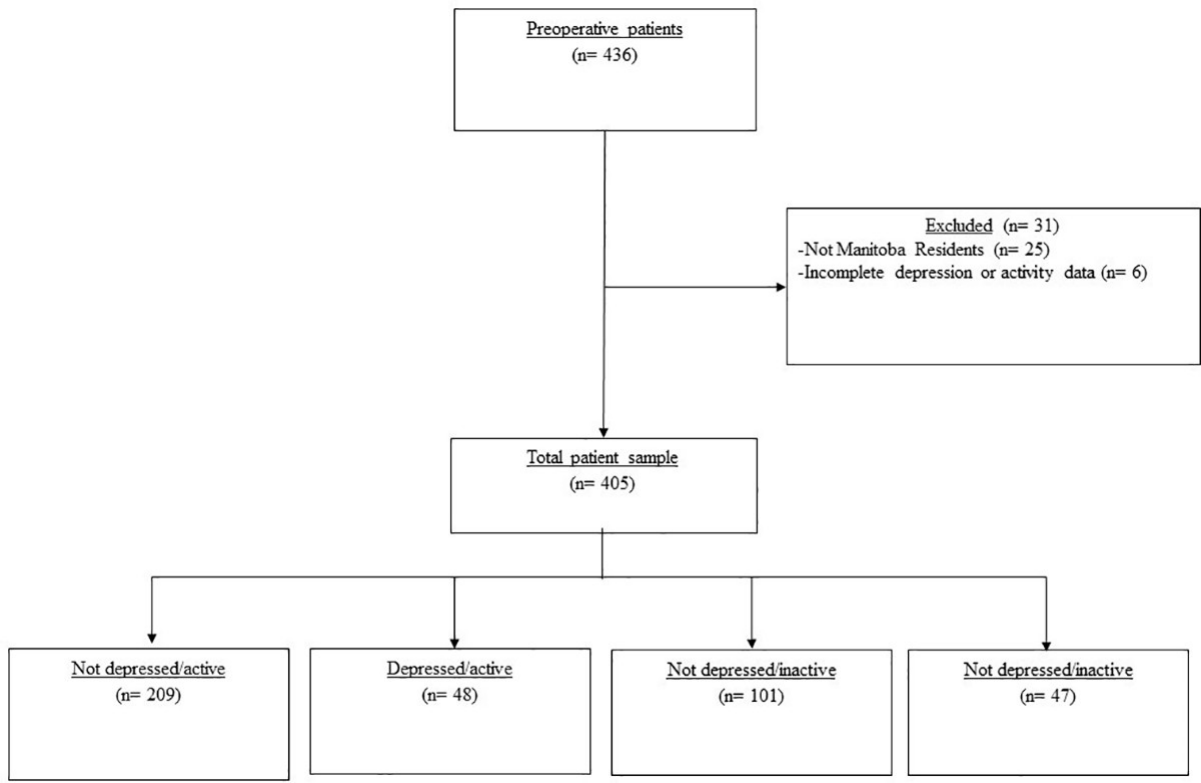
Study participant flow diagram. Description of how cardiac surgery patients in the study were stratified into the four groups categorized by depressive symptoms and physical activity behavior.

The number of patients who were eligible and approached for the study has been described previously [4, 5]. Examination of patients with complete IPAQ and PHQ-9 data resulted in a final sample of 405 participants for the secondary analysis (Fig 1). After stratifying patients by depressive symptoms and physical activity, 209/405 (52%) were Not depressed/Active, 48/405 (12%) were Depressed/Active, 101/405 (25%) were Not depressed/Inactive, and 47/405 (12%) were Depressed/Inactive (Fig 1 and Table 1). Significant differences in pre- and peri-operative characteristics between groups were found for previous arrhythmias, preoperative ejection fraction and HLOS (Table 1).

**Table 1:** Baseline characteristics by the combination of preoperative depression and physical activity.

Variable	Not depressed/ Active (n = 209)	Depressed/Active (n = 48)	Not depressed/ Inactive (n = 101)	Depressed/ Inactive (n = 47)	P-value
Preoperative					
Age	67(59–73)	63(58–68)	69(59–76)	66(60–74)	0.11
Sex (% female)	51(24%)	17(35%)	36(36%)	17(36%)	0.10
Operative status					0.14
Elective	155(75%)	35(73%)	64(64%)	29(62%)	
Urgent	53(25%)	13(27%)	36(36%)	18(38%)	
Winnipeg Residence	129(62%)	26(54%)	49(49%)	24(51%)	0.15
Preoperative ejection fraction	60(53–60)	60(48–60)	60(53–60)	53(38–60)	0.007
CCS class ≥3	106(51%)	25(52%)	58(57%)	34(72%)	0.05
NYHA class ≥3	40(19%)	16(33%)	26(26%)	14(30%)	0.11
Preoperative creatinine (umol/L)	77(65–91)	69(59–85)	76(64–94)	79(65–101)	0.12
Smoker	51(25%)	11(23%)	28(28%)	13(28%)	0.89
Hyperlipidemia	159(76%)	33(69%)	83(83%)	39(83%)	0.18
Hypertension	155(74%)	38(79%)	81(81%)	41(87%)	0.20
Type 2 Diabetes	71(34%)	16(33%)	31(31%)	17(36%)	0.93
Peripheral vascular disease	32(15%)	6(13%)	17(17%)	9(19%)	0.82
Previous myocardial infarction	72(34%)	13(27%)	42(42%)	20(43%)	0.25
Previous arrhythmia	25(12%)	9(19%)	8(8%)	11(23%)	0.04
PHQ-9 depression scores	2(0–3)	10(7–13)	3(1–6)	9(7–12)	<0.001
IPAQ MET-min/week	2079(1040–5508)	2033(1229–5457)	99(0–322)	65(0–297)	<0.001
IPAQ moderate-vigorous min/week	120 (0–420)	120(0–390)	0(0–0)	0(0–0)	<0.001
Operative					
Isolated CABG Procedure	118(56%)	27(56%)	59(58%)	31(66%)	0.68
Perioperative characteristics					
Cardio-pulmonary bypass time (minutes)	100(79–156)	89(70–122)	97(79–132)	87(66–130)	0.11
ICU length of stay (days)	1(1–2)	1(1–2)	1(1–2)	1(1–2)	0.64
Length of hospital stay (days)	7(5–11)	7(5–11)	8(6–12)	11(6–16)	0.03

Values are median (interquartile range) for continuous variables and frequency (percent) for categorical variables. P-values were calculated using a Chi-square or Kruskal-Wallis test. CCS, Canadian Cardiovascular Society. NYHA, New York Heart Association. CVA, cerebrovascular accident. TIA, transient ischemic attack. PHQ-9, Patient Health Questionnaire-9. IPAQ, International Physical Activity Questionnaire. CABG, coronary artery bypass graft. ICU, intensive care unit.

### Hospital length of stay

The multivariable linear regression models indicate that preoperative depressive symptoms and physical activity were not independently associated with operative HLOS when examined individually (Table 2). When comparing the depression/physical activity groups, there were no significant differences for HLOS.

**Table 2:** Multivariable linear regression analysis comparing physical activity and depressive symptoms in isolation and in combination and their association with log transformed length of hospital stay.

Variable	Beta Coefficient	Standard Error	P-Value
**Model 1: Physical activity and depression examined independently**			
Physically Inactive	0.047	0.050	0.35
Depressed	0.059	0.058	0.31
Model 2: Combination of physical activity and depression*			
Not Depressed/Active	-0.105	0.080	0.19
Depressed/Active	-0.068	0.099	0.49
Not Depressed/Inactive	-0.073	0.086	0.40

Mode1: Adjusted for age, sex, urban/rural residence, non-isolated coronary artery bypass, urgency status, diabetes, peripheral vascular disease, myocardial infarction, arrhythmia, hypertension, smoking status, cardio-pulmonary bypass time, and Acute Physiology and Chronic Health Evaluation score, Canadian Cardiovascular Society Classification, and Ejection Fraction.

Model 2: Adjusted for age, sex, urban/rural residence, non-isolated coronary artery bypass, urgency status, diabetes, peripheral vascular disease, myocardial infarction, arrhythmia, hypertension, smoking status, cardio-pulmonary bypass time, and Acute Physiology and Chronic Health Evaluation score, Canadian Cardiovascular Society Classification, and Ejection Fraction.

Missing covariate values were handled via multiple imputation

*Reference category for model 2 is the Depressed/Inactive group

### Re-hospitalization and mortality

**Fig 2:**
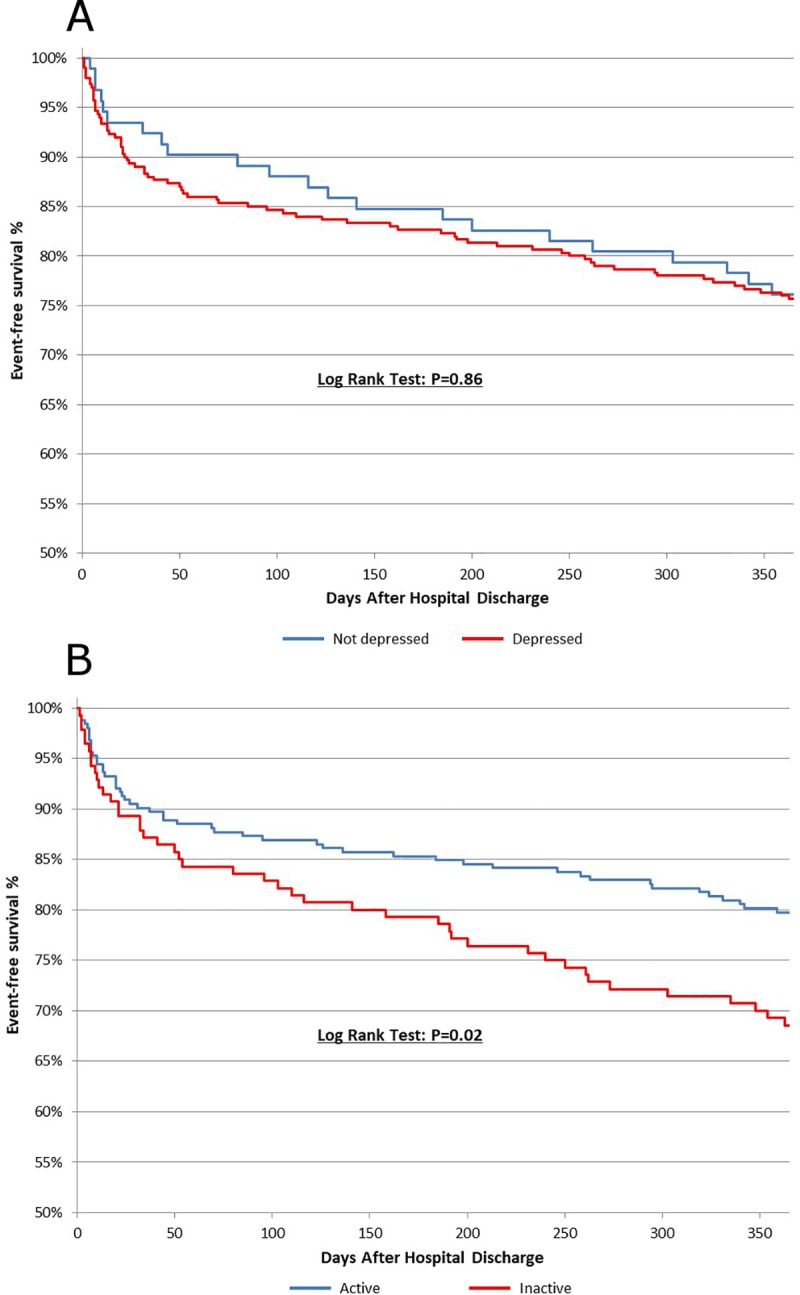
Kaplan-meier estimates for the composite outcome of one year re-hospitalization and mortality by physical activity status and depression status. **Panel A: Depression groups.** Blue = Not depressed, Red = depressed. **Panel B: Physical activity groups.** Blue = Active, Red = Inactive.

**Fig 3:**
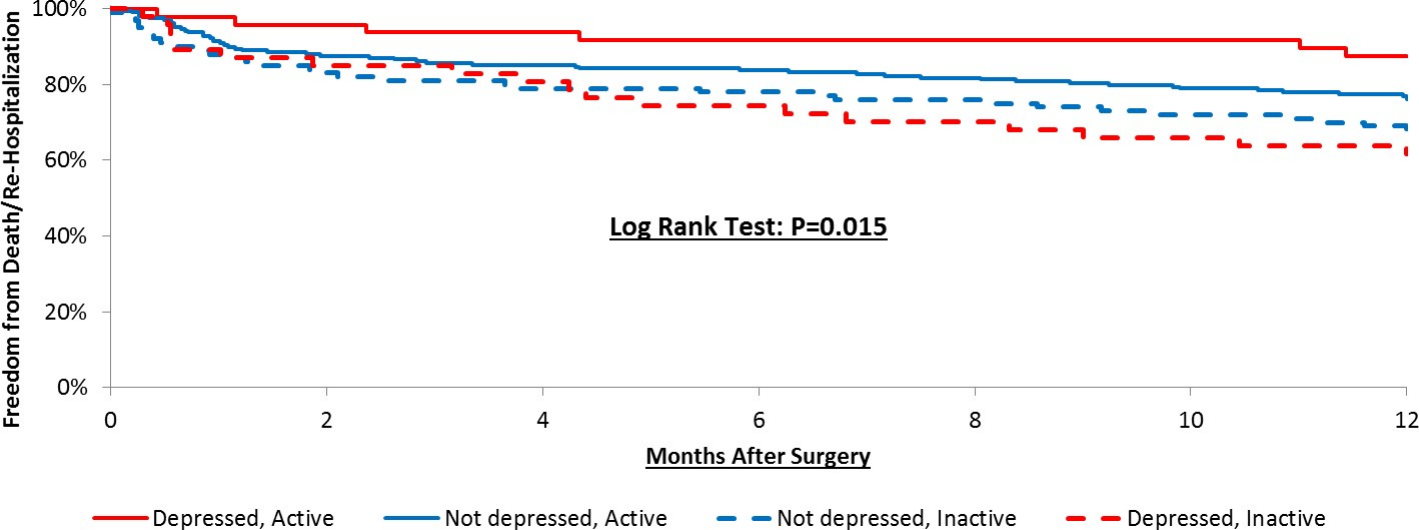
Kaplan-meier estimates for the composite outcome of one year re-hospitalization and mortality by depression/physical activity groups. Solid red = Depressed/Active group; Solid blue = Not depressed/Active group; Dashed blue = Not depressed/Inactive group; Dashed red = Depressed/Inactive group.

The most responsible causes based on ICD-10-CM codes were heart failure, complications due to surgical procedures, pleural effusion, and convalescence (S1 Table). Kaplan-Meier Curves for the individual, unadjusted associations of depressive symptoms and physical activity with the combined endpoint of re-hospitalization and mortality can be viewed in Fig 2. Preoperative depressive symptoms were not associated with the composite endpoint (Fig 2, Panel A). In contrast, patients who were defined as active prior to cardiac surgery had significantly lower rates of re-hospitalization/mortality one year postoperatively (Fig 2, Panel B). The Kaplan-Meier unadjusted estimates for the composite outcome were 76.1%, 87.5%, 68.0%, and 61.7% in the Not depressed/Active, Depressed/Active, Not depressed/Inactive, and the Depressed/Inactive groups, respectively (Fig 3; p = 0.02).

The Cox Proportional Hazards Regression models for the individual and combined associations for preoperative depressive symptoms and physical activity on one-year re-hospitalization and mortality can be viewed in Table 3. Preoperative depressive symptoms were not associated with the composite outcome. Whereas, patients defined as physically inactive before surgery had an independent, increased risk of re-hospitalization and mortality one-year postoperatively (p = 0.02). When depression and physical activity status were investigated in combination, the data revealed that individuals who were Depressed/Active were at a significantly lower risk of the composite endpoint compared to those who were Depressed/Inactive (p = 0.03; Table 3). Individuals who were Not depressed/Active at baseline also had a lower risk of experiencing the composite endpoint compared to those Depressed/Inactive (HR = 0.61) however this difference was not statistically significant (p = 0.11).

**Table 3:** Proportional Hazards Analysis comparing physical activity and depressive symptoms in isolation and in combination and their association on the combined endpoint of one year re-hospitalization and mortality.

Variable	Hazard Ratio (95% CI)	P-Value
**Model 1: Physical activity and depression examined independently**		
Physically Inactive	1.60 (1.05–2.42)	0.03
Depressed	0.93 (0.57–1.51)	0.77
Model 2: Combination of physical activity and depression*		
Not Depressed/Active	0.64 (0.36–1.15)	0.14
Depressed/Active	0.35 (0.14–0.89)	0.03
Not Depressed/Inactive	0.89 (0.48–1.64)	0.70

Mode1: Adjusted for age, sex, urban/rural residence, non-isolated coronary artery bypass, urgency status, diabetes, peripheral vascular disease, myocardial infarction, arrhythmia, hypertension, smoking status, cardio-pulmonary bypass time, and Acute Physiology and Chronic Health Evaluation score, Canadian Cardiovascular Society Classification, and Ejection Fraction.

Model 2: Adjusted for age, sex, urban/rural residence, non-isolated coronary artery bypass, urgency status, diabetes, peripheral vascular disease, myocardial infarction, arrhythmia, hypertension, smoking status, cardio-pulmonary bypass time, and Acute Physiology and Chronic Health Evaluation score, Canadian Cardiovascular Society Classification, and Ejection Fraction.

Missing covariate values were handled via multiple imputation.

*Reference category for model 2 is the Depressed/Inactive group

## Discussion

The purpose of this study was to determine the individual and combined impact of preoperative physical activity and depressive symptoms on operative HLOS and one-year postoperative re-hospitalization and mortality in cardiac surgery patients. Neither depressive symptoms nor physical activity were associated with HLOS when examined individually. Patients defined as Not depressed/Active prior to cardiac surgery had a shorter HLOS compared to those who were Depressed/Inactive. Preoperative physical activity, but not depressive symptoms, were independently associated with a lower risk of the composite endpoint of re-hospitalization/mortality. Examined in combination, the Depressed/Active group were more likely to have event-free survival one-year postoperatively, compared to the Depressed/Inactive group. Collectively, these data suggest that preoperative physical activity is an important indicator of perioperative health outcomes, and may have its greatest health benefits in those with elevated depressive symptoms post-cardiac surgery.

### Hospital length of stay

Our data suggests that neither self-reported physical activity nor elevated depressive symptoms were associated with HLOS following cardiac surgery, when examined in isolation or in combination after adjustment for a number of covariates (Table 2). Data concerning the benefit of preoperative physical activity behaviors [22, 23] and the impact of depression [24, 25] on post-cardiac surgical HLOS are conflicting, and studies which have demonstrated associations between HLOS. It is likely that the severity of symptoms that may limit functional capacity of patients including a low preoperative ejection fraction and worse CCS class are precluding patients from being physically active prior to their operation and contributing to the somatic symptoms of depression. It is also possible that patients are misreporting their physical activity levels and depressive symptoms prior to cardiac surgery [5]. To elucidate the impact of physical activity and depression on hospital outcomes, future studies use objective measures of physical activity and use a healthcare provider administered depression screening or assessment tool.

### One-year re-hospitalization and mortality

The present study demonstrated that patients defined as physically active prior to cardiac surgery were at a significantly lower risk of the composite outcome of one-year postoperative re-hospitalization/mortality. In contrast, there was no association between pre-surgical depressive symptoms and this composite outcome. Previous investigations in the cardiac surgery patient population have not considered preoperative physical activity in mediating the relationship between re-hospitalizations and other poor outcomes in depressed patients [2, 3, 26, 27]. Our study suggests that higher amounts of preoperative physical activity was protective of event-free survival (i.e., re-hospitalizations and mortality) one year postoperatively in patients who were depressed at baseline (Table 3). In fact, other investigations in non-cardiac surgery patients suggest that physical activity mediates the association between depression and cardiac-related morbidity and mortality [12, 13].

While there was a signal towards a protective association from the composite endpoint in the Not depressed/Active group compared to the Depressed/Inactive group, there was not a statistically significant difference between the groups (p = 0.11). These data suggest that physical activity is the main driver behind patients experiencing poor health outcomes postoperatively. It is also possible that the somatic symptoms of depression (e.g., feeling tired, slowness) were more affected by preoperative physical activity levels in the Depressed/Active group compared to the other depression/physical activity groups; somatic versus cognitive symptoms of depression may have a greater negative impact on cardiac-related health outcomes [28]. However, this possibility was not explored in the present study. Even so, our findings suggest that physical activity may have its most potent health effects in cardiac patients who are depressed.

### Future research

Our data suggest that a more physically lifestyle prior to cardiac surgery might be an important prognostic indicator pre- and postoperatively. As such, these findings support the implementation of preoperative rehabilitation (i.e., Prehab) programs designed to promote an active lifestyle so as to improve the health and overall quality of life in cardiac surgery patients [15, 16, 29, 30]. However, future investigations are needed to determine if Prehab is a sufficient stimulus to reduce depressive symptoms preoperatively.

Future investigations should also determine if changes in pre- and postoperative physical activity behaviors and depressive symptoms are associated with longer term postoperative health outcomes in cardiac surgery patients. A previous study suggests a long-term protective association with physical activity behaviors with mortality in cardiovascular disease patients with high levels of depressive symptoms [13]. By extension, it is possible that preoperative physical activity in cardiac surgery patients could translate into longer-term health outcomes, especially if a patient adopts a more physically active lifestyle pre- and postoperatively, but this possibility remains to be investigated. Importantly, postoperative assessment of physical activity following cardiac surgery may also provide insight into return of normal functioning of the patient before they required their surgical intervention [31].

More research is needed in the pre- and postoperative cardiac surgery period which use objective assessments of movement behaviors (e.g., accelerometry). These data would provide more accurate estimates of the impact of physical activity behaviors on post-cardiac surgical outcomes. Furthermore, it would be valuable to know if not only preoperative objectively assessed physical activity behaviors, but also sedentary behaviors, are independent predictors of postoperative health outcomes following cardiac surgery. Sedentary behaviors are unique from physical activity, and are defined as any waking behavior characterized by low energy expenditure (<1.5 METs) while in a seated or lying position [32]. These data would be important to researchers and healthcare providers who deliver standard care to cardiac surgery patients, to identify the amount of pre- and postoperative physical activity, or the reduction in sedentary time, needed to improve the outcomes of cardiac surgery patients.

### Limitations

There are some limitations to consider with the present study. The self-reported measure of physical activity behavior (IPAQ) may have resulted in an overestimation of the amount and intensity of weekly physical activity when compared with objectively measured physical activity [5]. However, patients may have also been limiting their physical activity levels because of their cardiac symptoms or have been told by their healthcare providers to limit their normal activity. The PHQ-9 is a depression screening tool and cannot be used to diagnose major depressive disorder. Strengths of the PHQ-Q are its feasibility of implementing in clinical practice, its good diagnostic accuracy for any depressive disorder in patients with coronary artery disease [33]. Patients may have also over reported their preoperative depressive symptoms because they may have dwelled on possible negative surgical outcomes. Other mental health disorders worth investigation preoperatively such as anxiety and post-traumatic stress disorder, were not considered in this study, and are known to influence health outcomes after cardiac surgery and have overlapping symptoms with depression [34]. This analysis does not consider relief or worsening of depressive symptoms postoperatively; changes in depression immediately postoperatively are known to influence postoperative health outcomes [2, 35]. However, the intention of the present study was to examine preoperative depressive symptoms (and physical activity behavior) as a single entity and not postoperative stress responses.

## Conclusion

This study provides novel insights into the impact of preoperative physical activity behavior on the postoperative health outcomes or cardiac surgery patients. A more physically active lifestyle prior to cardiac surgery may have its most beneficial effects in patients with elevated depressive symptoms. As such, the findings from this study suggest that preoperative physical activity behavior should be considered in the evaluation of short-term health outcomes among patients undergoing a cardiac surgical intervention.

## Supporting information

S1 Table Common most responsible ICD-10-CM diagnosis code for hospital readmission. (DOCX)
