# Supplementary material for: Impact of preoperative physical activity and depressive symptoms on post-cardiac surgical outcomes
Source: PLoS One. 2019 Feb 28;14(2):e0213324. doi: 10.1371/journal.pone.0213324 (PMC6394976; doi:10.1371/journal.pone.0213324)
Supplement: S1 Table — (DOCX) [file pone.0213324.s001.docx]

| **Most Responsible ICD-10-CM Code** | **Percentage of Readmissions** |
| --- | --- |
| I50 – Heart Failure | 8.3% |
| T81 – Complications of Procedures, Infection | 7.0% |
| J90 – Pleural Effusion, Not Elsewhere Classified | 5.7% |
| Z54 - Convalescence | 4.5% |

**Supplementary table 1.** Common “Most Responsible” ICD-10-CM diagnosis code for hospital readmission within 1 year.
